# Factors influencing the impact of oral health on the daily activities of adolescents, adults and older adults

**DOI:** 10.11606/S1518-8787.2018052000042

**Published:** 2018-04-04

**Authors:** Jaqueline Vilela Bulgareli, Eduardo Tanajura de Faria, Karine Laura Cortellazzi, Luciane Miranda Guerra, Marcelo de Castro Meneghim, Glaucia Maria Bovi Ambrosano, Antonio Carlos Frias, Antonio Carlos Pereira

**Affiliations:** I Universidade de Campinas. Faculdade de Odontologia de Piracicaba. Programa de Pós-Graduação em Odontologia. Piracicaba, SP, Brasil; II Universidade de Campinas. Faculdade de Odontologia de Piracicaba. Programa de Pós-Graduação Mestrado Profissional em Odontologia em Saúde Coletiva. Piracicaba, SP, Brasil; III Universidade de Campinas. Faculdade de Odontologia de Piracicaba. Departamento de Odontologia Social. Piracicaba, SP, Brasil; IV Universidade de São Paulo. Faculdade de Odontologia. Departamento de Odontologia Social e Preventiva. São Paulo, SP, Brasil

**Keywords:** Oral Health, Socioeconomic Factors, Quality of Life, Dental Health Surveys, Saúde Bucal, Aspectos Socieconômicos, Qualidade de Vida, Inquéritos de Saúde Bucal

## Abstract

**OBJECTIVE:**

Analyze if clinical, sociodemographic and access to dental services variables influence the impact of oral health on the daily activities of adolescents, adults and older adults.

**METHODS:**

A cross-sectional study with secondary data from the State Oral Health Survey (SB São Paulo 2015) conducted in 163 cities of São Paulo. A total of 17,560 individuals from three age groups: 15–19-year-old (n = 5,558), 35–44-year-old (n = 6,051), and older people of 65 years or more (n = 5,951) participated in the survey. The selection was made by probabilistic sample by conglomerates in two stages. The endpoint variable was the impact of oral health on daily activities, evaluated by the Oral Impacts on Daily Performances questionnaire, containing questions about eating, talking, oral hygiene, relaxation, sports practice, smile, study or work, social contact, and sleep. Oral Impacts on Daily Performances was dichotomized with and without impact. The independent variables were sociodemographic, clinical and access variables, divided into three blocks. A hierarchical multiple logistic regression analysis was performed considering the complex sampling plan of clusters. Each observation received a specific weight, depending on the location that resulted in weighted frequencies and adjusted for the design effect.

**RESULTS:**

The presence of oral health impact was observed in 27.9% of the individuals. In block 1, female gender and black/brown ethnic group had a greater chance of impact of oral health on quality of life, as well as the adults and the older adults in relation to adolescents. In block 2, family income up to R$1,500 was associated with the presence of impact. In block 3, individuals who reported toothache, used the public service and sought dental treatment had a greater chance of impact.

**CONCLUSIONS:**

Sociodemographic, clinical and access to health services variables influence the impact of oral health on the daily activities of adolescents, adults and older adults.

## RESUMO

**OBJETIVO:**

Analisar se variáveis clínicas, sociodemográficas e de acesso aos serviços odontológicos influenciam o impacto da saúde bucal nas atividades diárias de adolescentes, adultos e idosos.

**MÉTODOS:**

Estudo transversal com dados secundários da Pesquisa Estadual de Saúde Bucal (SB São Paulo 2015) realizada em 163 municípios de São Paulo. Participaram do inquérito 17.560 indivíduos de três grupos etários: adolescentes de 15–19 anos (n = 5.558), adultos de 35–44 anos (n = 6.051) e idosos de 65 anos ou mais (n = 5.951). A seleção foi feita por amostra probabilística por conglomerados em dois estágios. A variável desfecho foi o impacto da saúde bucal sobre as atividades diárias, avaliado pelo questionário *Oral Impacts on Daily Performances*, contendo questões sobre comer, falar, higiene bucal, relaxamento, prática esportiva, sorriso, estudo ou trabalho, contato social e sono. O *Oral Impacts on Daily Performances* foi dicotomizado em com e sem impacto. As variáveis independentes foram as sociodemográficas, clínicas e de acesso, divididas em três blocos. Realizou-se análise de regressão logística múltipla hierarquizada considerando o plano amostral complexo de conglomerados. Cada observação recebeu um peso específico, dependendo da localização que resultou em frequências ponderadas e ajustadas para o efeito do delineamento.

**RESULTADOS:**

A presença de impacto da saúde bucal foi constatada em 27,9% dos indivíduos. No bloco 1, o sexo feminino e o grupo étnico preto/pardo tiveram maior chance de impacto da saúde bucal na qualidade de vida, bem como os adultos e idosos em relação aos adolescentes. No bloco 2, a renda familiar até R$1.500 teve associação com a presença de impacto. No bloco 3, os indivíduos que relataram dor de dente, frequentaram o serviço público e procuraram tratamento odontológico tiveram maior chance de impacto.

**CONCLUSÕES:**

As variáveis sociodemográficas, clínica e de acesso ao serviço de saúde influenciam o impacto da saúde bucal nas atividades diárias de adolescentes, adultos e idosos.

## INTRODUCTION

There has been growing recognition of oral problems as having a negative impact on the performance of daily activities and, consequently, on individuals’ quality of life. In fact, besides pain and suffering, oral diseases and their disorders can also cause social deprivation and psychological embarrassment^21^.

The most used indexes for determining actions, strategies and programs in oral health mainly consider biological factors, not taking into account the understanding already established in the literature on the importance and impact of psychological and social factors on oral diseases^2^.

Studies have found a strong association between oral problems and negative impact on the quality of life of individuals. Disorders such as caries and dental pain have caused functional, social and psychological adverse effects^10^ ^,^ ^25^.

However, even with the advancement of research on the impact of oral conditions on quality of life, these studies are usually restricted to analyzing specific populations, such as schoolchildren or institutionalized elders, and there are few populational surveys in the literature^10^ ^,^ ^21^.

The National Oral Health Survey (SB Brazil 2010), carried out by the Ministry of Health in an innovative and pioneering way, allowed the collection of information capable of promoting an analysis of the impact of oral health on people's quality of life and served as the basis for a state oral health research project named SB São Paulo, 2015.

According to estimates by the Brazilian Institute of Geography and Statistics (IBGE), the state of São Paulo has a population that corresponds to approximately 21% of the total population of Brazil. There are approximately 42 million people and, of these, about 80% are over 15 years of age^12^.

Epidemiological surveys make it possible to identify the needs of the population, in addition to existing health and disease conditions, allowing them to be quantified, as well as instrumentalizing the planning and organization of oral health services^26^.

Thus, knowing the perception and behavior of the individual in relation to their oral health, as well as the influence and impact of oral health on their daily activities, offers subjective data, besides measurable and quantitative data. It can thus be of great value for the planning, definition, and organization of oral health care services and programs.

Thus, the objective of this study was to analyze whether clinical, sociodemographic and access to dental services variables influence the impact of oral health on the daily activities of adolescents, adults and older adults.

## METHODS

### Type of Study

Cross-sectional study with secondary data from the State Oral Health Survey (SB São Paulo 2015 Project) conducted in 163 cities in the state of São Paulo.

### Sample Characterization

This was a state-based survey, with representation for six regions, representing the entire state of São Paulo (São Paulo Capital, Metropolitan Region of São Paulo and the Regional Health Departments – DRS-2 to 17). For this, 178 cities plus the state capital were drawn (primary sampling units). In the second stage, 390 census tracts were selected (two sectors for 178 cities and 36 sectors for the city of São Paulo). The sample plan was prepared by a conglomerate in two stages of a lottery with probability proportional to the size of the population. The exhaustion technique was used with a minimum sample size for each primary sampling unit, where all households in the census tract were visited following the planned route, and the individuals of the age groups of the study were examined. As it was not possible to carry out examinations in all cities randomly selected (primary sampling units) in the respective domains, as well as in some census tracts, the sampling fractions were corrected taking into account the non-response rates for each of the stages of the draw. Absentees and those who refused to participate in the study were excluded, totaling 17,560 people examined in 163 cities for the three age groups, with 5,551, 6,051, and 5,951 individuals, respectively, for the age groups of adolescents aged 15–19 years, adults 35–44 years old, and 65 years old or older^20^.

### Training and Calibration of Examiners

The team calibration process lasted at least 24 hours of work, contemplating the theoretical and practical aspects of the indexes used. The percentage of intra and inter-examiner agreement was checked in order to verify the reproducibility of the study^20^.

Epidemiological examinations and interviews were performed in the homes of the volunteers by dental surgeons previously trained and calibrated by the consensus technique. The minimum acceptable value of kappa for each examiner, age group, and disorder studied was 0.65^17^.

Oral examinations were performed to evaluate the prevalence and severity of caries, periodontal disease, malocclusion, and use or need for a prosthesis. In addition, a questionnaire was applied to individuals examined at home, which included questions regarding socioeconomic characterization, the use of dental services and self-reported oral morbidity, self-perception of oral health, and social capital. For this study, the methodology used in SB São Paulo 2015 followed the recommendations of the WHO in the 4th edition of its Instruction Manual for Basic Epidemiological Survey in Oral Health^26^, with the appropriate adjustments expressed in the SB Brazil Project 2010^17^.

### Study Variables

The endpoint variable was the Impact of Oral Impacts on Daily Performances (OIDP). This variable was evaluated by the instrument Oral Impacts on Daily Performances^1^ (OIDP). The instrument consists of nine questions of daily performances, such as eating, speaking, oral hygiene, relaxation, sports practice, smile, study and work, social contact, and sleep. Each item was preceded by the question “Some people have problems that may have been caused by their teeth. From the situations below, which ones apply to you in the last six months?”. The answer options were: no (code 0), yes (code 1) and do not know or did not want to respond (code 9). Code 9 was treated as missing information for each OIDP question. The simple counting of the scores was by means of nine variables (yes or no). The OIDP was dichotomized with and without impact, and the presence of impact on the volunteer's daily activities was characterized by the “yes” answer in at least one question.

The independent variables were grouped into three blocks: block 1 included the variables gender (male, female), age group (15–19 years, 35–44 years, ≥ 65 years) and ethnic group (white; black or brown). Block 2 approached the family income (dichotomized by the median up to R$1,500 and > R$1,500). Block 3 included tooth pain (no, yes), dental attendance (< 1 year, 1 year or more), type of service used (public, others), the reason for consultation (review or prevention, treatment) and CPOD index (dichotomized by the median in CPOD≤15 and CPOD>15). These variables were organized following the conceptual model adapted from Peres et al.^21^, in which the author uses a hierarchical model for multiple analysis of the potential predictors for OIDP. The independent variables are introduced in levels, from the most distal to the most proximal in relation to the endpoint. In the present study, block 1 presents the most distal demographic variables in relation to the outcome variable; block 2, the socioeconomic variable; and in block 3, the variables of access to dental services and diseases and oral disorders, closer to the endpoint variable (Figure).

**Figure:**
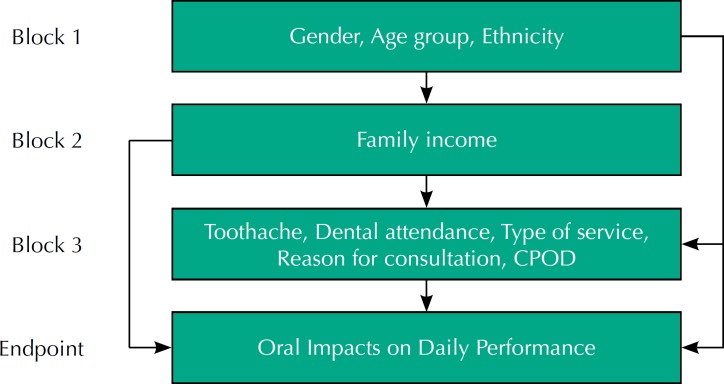
Conceptual model for the study of the association between independent variables and the impact of oral health on daily activities (adapted from Peres et al.^21^). CPOD: index of permanent, decayed, missing and filled teeth

### Data Analysis

For the data analysis, the association between the OIDP and the independent variables was evaluated through a hierarchical multiple logistic regression model. The data analysis was performed by PROC SURVEYFREQ and PROC SURVEYLOGISTIC procedures, considering the complex sample plan of conglomerates. Each observation received a specific weight, depending on the location that resulted in weighted frequencies and adjusted for the design effect. The variables with p ≤ 0.20 of each block were tested in the multiple logistic regression model, remaining in the model those that continued to be associated with the OIDP with p ≤ 0.05 after adjusting for the variables of the same block and to the hierarchically superior ones.

### Ethical Aspects

Because this was a study involving secondary data and already made public and unrestricted access to the state epidemiological survey of oral health (SB São Paulo 2015), it was waived by the Ethics and Research Committee of Faculdade de Odontologia de Piracicaba (FOP) UNICAMP according to document CEP/FOP 42/2016. A free and informed consent form was signed by all persons examined in the survey^20^.

## RESULTS

Of the 17,560 individuals in the state survey sample, 16,776 individuals actually participated in this study, 5,402 (32.2%), 5,834 (34.8%) and 5,540 (33.0%) individuals, respectively, for the age groups of 15–19, 35–44 and 65 years or more. The difference of the participants in relation to the total number of the sample studied was due to the exclusion of 784 individuals who failed to respond to one or more OIDP items. The presence of an impact on oral health was verified in 4,695 (27.9%) individuals (1,134, 2,152, and 1,409 individuals, respectively for the age groups of 15–19, 35–44, and 65 years or more) (Table 1).

**Table 1:** Distribution of OIDP impact frequencies according to the analyzed variables. SB São Paulo, 2015.

Variable	Category	n	%	OIDP			
				No impact		Impact	
				Frequency	%	Frequency	%
Block 1							
Gender	Male	6,306	37.6	4,897	77.7	1,409	22.3
	Female	10,470	62.4	7,184	68.6	3,286	31.4
Age group (years)	15–19	5,402	32.2	4,268	79.0	1,134	21.0
	35–44	5,834	34.8	3,682	63.1	2,152	36.9
	≥ 65	5,540	33.0	4,131	74.6	1,409	25.4
Ethnic group	White	10,647	63.5	7,988	75.0	2,659	25.0
	Black/Brown	6,129	36.5	4,093	66.8	2,036	33.2
Block 2							
Income	Up to R$1,500	6,843	46.5	4,490	65.4	2,353	34.6
	> R$1,500	7,863	53.5	6,001	74.8	1,862	25.2
Block 3							
Toothache	No	8,152	70.9	6,510	77.6	1,642	22.3
	Yes	3,354	29.1	1,414	42.7	1,940	57.3
Dental attendance	< 1 year	7,992	50.3	5,662	70.2	2,330	29.8
	1 year or more	7,884	49.7	5,798	69.8	2,086	30.2
Type of dental service	Public	6,452	40.5	4,392	65.0	2,060	35.0
	Others	9,475	59.5	7,183	73.8	2,292	26.1
Reason for dental consultation	Revision/Prevention	4,277	27.0	3,694	85.0	583	15.0
	Treatment	11,541	73.0	7,850	65.4	3,691	34.6
CPOD	≤ 24	8,408	50.1	6,316	72.1	2,092	27.9
	> 15	8,368	49.9	5,765	68.9	2,603	31.1

OIDP: oral impacts on daily performances; SB: dental health; CPOD: index of permanent, decayed, missing and filled teeth

Table 2 shows the results of the individual analyzes and the final model of the hierarchical multiple logistic regression analysis. Among the independent variables of block 1, female gender had a greater chance (OR = 1.26, 95%CI 1.15–1.39, p < 0.0001) of OIDP when compared to males, as well as the adult age group (OR = 3.15, 95%CI 2.65–3.74, p < 0.0001) and older adults (OR = 1.48, 95%CI 1.24–1.78, p < 0.0148) in relation to adolescents. There was a greater chance of OIDP among black/brown ethnic group (OR = 1.32, 95%CI 1.17–1.48, p < 0.0001) than in whites, as well as among individuals presenting family income up to R$1,500 (OR = 1.30, 95%CI 1.08–1.56, p < 0.0001) inserted in block 2. Of the variables tested in block 3, individuals who reported tooth pain (OR = 3.76, 95%CI 3.37–4.19, p < 0.0001), who attended the public service (OR = 1.32; 95%CI 1.21–1.45; p < 0.0001), and the main reason for seeking care was dental treatment (OR = 2.28, 95%CI 1.97–2.64, p < 0.0001) had a greater chance of impact on oral health.

**Table 2:** Gross and adjusted odds ratios between the OIDP impact and the variables analyzed. SB São Paulo, 2015.

Variable	Category	OR_b_ (95%CI)	p	OR_a_ (95%CI)	p
Block 1					
Gender	Male	Ref		Ref	
	Female	1.59 (1.40–1.82)	< 0.0001	1.26 (1.15–1.39)	< 0.0001
Age group (years)	15–19	Ref		Ref	
	35–44	2.35 (2.06–2.70)	< 0.0001	3.15 (2.65–3.74)	< 0.0001
	≥ 65	1.12 (0.95–1.32)	< 0.0001	1.48 (1.24–1.78)	0.0148
Ethnic group	White	Ref		Ref	
	Black/Brown	1.45 (1.29–1.63)	< 0.0001	1.32 (1.17–1.48)	< 0.0001
Block 2					
Income	Up to R$ 1,500	1.57 (1.36−1.80)	< 0.0001	1.30 (1.08–1.56)	< 0.0001
	> R$ 1,500	Ref		Ref	
Block 3					
Toothache	No	Ref		Ref	
	Yes	4.66 (4.01–5.40)	< 0.0001	3.76 (3.37–4.19)	< 0.0001
Dental attendance	< 1 year	Ref			
	1 year or more	1.02 (0.92–1.12)	0.7350	-	
Type of dental service	Public	1.52 (1.35–1.71)	< 0.0001	1.32 (1.21–1.45)	< 0.0001
	Others	Ref		Ref	
Reason for dental consultation	Review	Ref		Ref	
	Treatment	3.01 (2.73–3.33)	< 0.0001	2.28 (1.97–2.64)	< 0.0001
CPOD	≤ 24	Ref			
	> 15	1.17 (1.05–1.29)	0.0031	-	

OR_b_ (95%CI): gross odds ratio with the confidence interval; OR_a_ (95%CI): adjusted odds ratio with the confidence interval; OIDP: oral impacts on daily performances; SB: dental health; CPOD: index of permanent, decayed, missing and filled teeth; Ref: reference

## DISCUSSION

From the sociodemographic variables studied, being female had a greater impact on the quality of life. This result corroborates the results found in other studies^16^ ^,^ ^21^ ^,^ ^25^. This difference between men and women can result from oral health problems in social, economic, cultural and historical contexts^16^. Borrell and Artazcoz^4^ report that, for a long time, women have developed a cultural role of family responsibility and care; therefore, they would be more concerned about their oral health. Men are less concerned about the impact of oral conditions on their quality of life, to the point of reporting the problem only when the condition is already well advanced, with physical alteration and pain, becoming more significant^4^ ^,^ ^16^. The result of the present study can also be attributed to the fact that women present greater demands on the aesthetic appearance of the smile, which makes them more sensitive to the presence of dental caries^5^, in addition to hormonal conditions and a higher prevalence of systemic diseases that can influence their oral health^9^.

Age is a major moderator of oral health self-perception and older individuals have a poorer quality of life-related to oral health. With increasing age, there is a perception of a continuous deterioration of the quality of life due to systemic, psychological and social factors^6^. Some authors have found significant differences between people aged 45 to 64 years and people over 65 years of age and have identified that the younger ones presented the greater impact of oral health in daily activities even with better oral conditions^13^. This indicates that individual needs and expectations can influence the degree of satisfaction with health^15^.

With aging, people tend to consider dental illnesses to be less significant because they believe that their health is deteriorating, secondary to general health problems^15^. In this study, the adults and the older adults were the age groups that presented the greatest chance of impact on the daily activities when compared to adolescents, corroborating other reports in the literature^8^ ^,^ ^9^ ^,^ ^18^. One hypothesis for this may be the greater need for dental treatment, given the greater difficulty of access to treatment^9^.

The black/brown ethnic group and low family income were associated with the outcome. In fact, other studies have found that sociodemographic disparities are determinant in the experience of oral diseases^7^, in addition to observing a correlation between black populations living in places with low human development index and the prevalence of dental caries, dental absences, and accumulated odontological treatment^10^. Peres et al.^21^ also observed this relationship in a study with adolescents, in which the impact on quality of life, influenced by oral health issues, was higher in brown and low-income populations. The inequality among ethnic groups in relation to the risk of caries has been associated with the worse socioeconomic status of black and mulatto people against whites^3^. This finding regarding the greater possibility of caries in brown and black adolescents is similar in another study that indicates a poorer health condition for non-whites, which points out the socioeconomic differences that some ethnic groups face^11^.

Low-income people are also more likely to report chewing problems and psychosocial changes in daily life than those with high income, even after considering the presence of oral complaints such as dental caries, periodontal disease, and tooth loss^14^. This research reinforces the results found in this study, where there was a greater impact of oral health on daily activities in lower income families.

Another variable related to the impact of oral health was the presence of dental pain. Toothache causes physical discomfort and can directly affect social contact, in addition to diminishing the functional capabilities of the teeth. Other studies have found toothache^21^ and lack of teeth^24^ as the main problem impacting oral health.

Regarding access to the dental service, it is important to recall that the history of Brazilian dental care was marked for a long time by basic curative actions in its express majority, in addition to the restricted access of the population to public oral health services for schoolchildren. The rest of the population was usually treated in emergency services, where the conduct was usually dental extraction^19^. This generated a population with a high rate of edentulism and a culture of which the public dental service was of poor quality.

In the present study, the use of the public dental service was related to the impact of oral health on people's daily activities. This finding can be explained by the hypothesis that social vulnerability is directly related to the impact of oral health, where geographic and sociodemographic inequities are determinant in the experience of oral diseases^7^ and the greater demand for public services by the lower income population.

Even if differences in health needs are not eliminated only through the use of health services, access to quality services can improve poor health conditions in populations and, consequently, have a positive impact on the quality of life of individuals.

Therefore, it is necessary to reflect on the resolubility and quality of oral health services offered to the population, as opposed to the hypothesis of social vulnerability directly related to the worst impact of oral health on people's daily activities^22^.

In this study, the variable motive of the last dental consultation to perform treatment has had an impact on people's oral health. This is probably because those who seek the public service for dental treatment usually have accumulated demand for services of greater complexity and have a greater chance of having experienced some negative dental impact.

As a limitation of this study, the cross-sectional design is emphasized, and it is not possible to establish any type of causal relationship, which generates difficulties in asserting whether the associations presented precede or follow the occurrence of the result. However, the results presented here are reliable, since they were obtained in a probabilistic sample by clusters representative of the population of the state of São Paulo. In addition, the designeffect correction was used in data analysis, a procedure recommended for studies with complex samples^23^.

Information on the health conditions of the population and its determinants, as well as their needs and the patterns of use of health services, are of great relevance in guiding health policies. In addition, population-based epidemiological studies, as in the present study, can generate important strengthening of oral health surveillance in the region studied, since it contributes to the identification of the oral health impact of the studied age groups, through sociodemographic, clinic and access to oral health services factors.

Sociodemographic, clinical and access to health services variables influenced the impact of oral health on the daily activities of adolescents, adults and older adults.
